# Shaping of Natural Killer Cell Antitumor Activity by *Ex Vivo* Cultivation

**DOI:** 10.3389/fimmu.2017.00458

**Published:** 2017-04-26

**Authors:** Markus Granzin, Juliane Wagner, Ulrike Köhl, Adelheid Cerwenka, Volker Huppert, Evelyn Ullrich

**Affiliations:** ^1^Clinical Research, Miltenyi Biotec Inc., Gaithersburg, MD, USA; ^2^Division for Stem Cell Transplantation and Immunology, Department for Children and Adolescents Medicine, Hospital of the Goethe University, Frankfurt, Germany; ^3^LOEWE Center for Cell and Gene Therapy, Cellular Immunology, Goethe University, Frankfurt, Germany; ^4^Institute of Cellular Therapeutics, Integrated Research and Treatment Center Transplantation, Hannover Medical School, Hannover, Germany; ^5^Innate Immunity Group, German Cancer Research Center, Heidelberg, Germany; ^6^Division of Immunbiochemistry, Medical Faculty Mannheim, Heidelberg University, Heidelberg, Germany; ^7^R&D Reagents, Miltenyi Biotec GmbH, Bergisch Gladbach, Germany

**Keywords:** natural killer cells, natural killer cell cultivation, natural killer cell expansion, natural killer cell therapy, natural killer cell cytotoxicity, *ex vivo* stimulation

## Abstract

Natural killer (NK) cells are a promising tool for the use in adoptive immunotherapy, since they efficiently recognize and kill tumor cells. In this context, *ex vivo* cultivation is an attractive option to increase NK cells in numbers and to improve their antitumor potential prior to clinical applications. Consequently, various strategies to generate NK cells for adoptive immunotherapy have been developed. Here, we give an overview of different NK cell cultivation approaches and their impact on shaping the NK cell antitumor activity. So far, the cytokines interleukin (IL)-2, IL-12, IL-15, IL-18, and IL-21 are used to culture and expand NK cells. The selection of the respective cytokine combination is an important factor that directly affects NK cell maturation, proliferation, survival, distribution of NK cell subpopulations, activation, and function in terms of cytokine production and cytotoxic potential. Importantly, cytokines can upregulate the expression of certain activating receptors on NK cells, thereby increasing their responsiveness against tumor cells that express the corresponding ligands. Apart from using cytokines, cocultivation with autologous accessory non-NK cells or addition of growth-inactivated feeder cells are approaches for NK cell cultivation with pronounced effects on NK cell activation and expansion. Furthermore, *ex vivo* cultivation was reported to prime NK cells for the killing of tumor cells that were previously resistant to NK cell attack. In general, NK cells become frequently dysfunctional in cancer patients, for instance, by downregulation of NK cell activating receptors, disabling them in their antitumor response. In such scenario, *ex vivo* cultivation can be helpful to arm NK cells with enhanced antitumor properties to overcome immunosuppression. In this review, we summarize the current knowledge on NK cell modulation by different *ex vivo* cultivation strategies focused on increasing NK cytotoxicity for clinical application in malignant diseases. Moreover, we critically discuss the technical and regulatory aspects and challenges underlying NK cell based therapeutic approaches in the clinics.

## Introduction

As an important part of the innate immune system, natural killer (NK) cells are deployed as first line of defense against aberrant cells caused by viral infections or malignancies. Human NK cells can be identified *via* their morphology as large granular lymphocytes, and *via* their surface marker profile, as they express by definition CD56, but not CD3. The NK cell compartment can be further divided into subpopulations. There are two main NK cell subsets that can be distinguished, the CD56^high^CD16^neg^ subpopulation, which has mostly immune modulatory function, mainly accomplished by interferon (IFN)-γ secretion, and the CD56^low^CD16^pos^ fraction with direct cytotoxic capacity (1–3). NK cell activation is based on a balanced system integrating signals from activating and inhibitory receptors. Inhibitory signals derive mainly from germ-line encoded inhibitory killer cell immunoglobulin-like receptors (KIRs). Ligands for inhibitory KIRs, in humans major histocompatibility complex (MHC) class I molecules, are highly expressed by healthy cells and thereby prevent NK cell activation. Malignant cells often downregulate MHC class I molecules on their surface to evade T cell attack (4). However, these so-called “missing-self” cells are recognized by NK cells through inhibitory receptors, and as signals from activating receptors prevail, NK cells become active and react against the encountered targets. Alternatively, NK cells can be activated by overexpression of stress-induced surface ligands on infected or abnormal cells, which are recognized by activating receptors, such as the natural cytotoxicity receptors (NCRs) NKp30, NKp44, and NKp46, and the so-called C-type lectin-like receptors, such as NKG2D (1, 5–9). In this case, activating signals outbalance inhibitory self-signals and lead to NK cell activation. Furthermore, NK cells become activated upon encounter of antibody-coated targets by CD16, which binds to the Fc portion of the antibody and mediates a strong activating signal. By means of activating and inhibitory receptors, NK cells, unlike T and B-lymphocytes, can react immediately without prior priming or antigen presentation.

Activated NK cells execute effector functions through different mechanisms. NK cells mediate direct cytotoxicity *via* the exocytosis pathway with release of cytotoxic granules, which contain granzymes and perforin, resulting in lysis of the target cell (10). In addition, NK cells induce apoptosis of target cells by expression of death receptor ligands, such as Fas ligand or tumor necrosis factor-related apoptosis-inducing ligand (TRAIL) (11). Production and release of IFN-γ by NK cells after activation also has multiple functional consequences, with particular relevance in tumor surveillance, as IFN-γ inhibits tumor angiogenesis, has antimetastatic activity, and acts pro-apoptotic (12, 13).

The ability of tumor cells to bypass the immune response is a basic prerequisite for cancer formation and progression. Within immune editing, tumors undergo genetic, epigenetic, and phenotypic changes, thereby becoming a heterogeneous cell population that is hardly visible to or assailable by immune cells due to downregulation of tumor antigens and NCR ligands (14). Additionally, malignant cells suppress NK cells by blocking the NKG2D receptor *via* shedding of NKG2D ligands (15–17) or upregulation of inhibitory MHC class I molecules (18, 19). Immunosuppressive cytokines such as transforming growth factor-β, interleukin (IL)-10, or immunosuppressive enzymes, such as indoleamin 2,3-dioxigenase, further impair antitumor NK cell responses of cancer patients (20–22).

*Ex vivo* modulation of NK cell receptor expression is therefore an important tool to overcome immune response inhibition. A number of studies reported an upregulation of DNAM-1, NKG2D, and other NK cell-activating receptors under certain culture conditions, mostly involving stimulation by IL-2 (23–26). In addition, other ILs such as IL-12, IL15, IL-18, or IL-21 and Type I IFNs shape the NK cell receptor expression profile (27–31).

**Figure 1:**
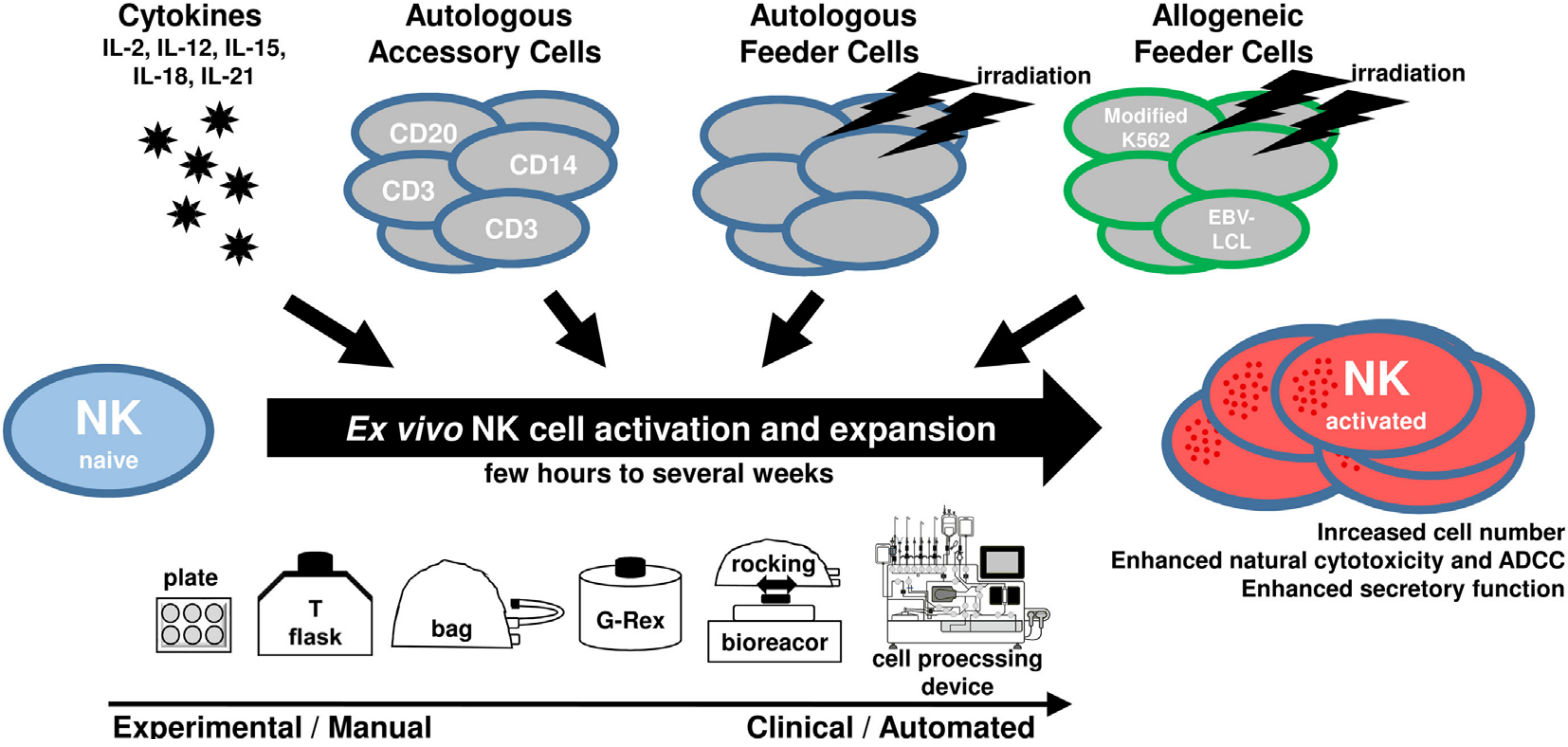
**Scheme showing main components utilized for *ex vivo* natural killer (NK) cell activation and expansion procedures**.

Natural killer cells can play an important role for cellular immunotherapy and the adoptive transfer of NK cells represents an attractive strategy to treat cancer patients (32, 33). In this context, *ex vivo* expansion of NK cells prior to their clinical application is not only required to increase the applicable cell doses but it is also reasonable to pre-activate and modify their antitumor features. For *ex vivo* cultivation, NK cells from different sources can be stimulated with different cytokines, and, to reach efficient expansion rates, NK cells are cultured among autologous accessory cells or together with different types of growth-inactivated autologous or allogeneic feeder cells (Figure 1). Of note, it is possible to genetically engineer NK cells *ex vivo* to further augment their antitumor activity, for example, to integrate chimeric antigen receptors against distinct tumor antigens (34, 35). In this review, we focus on the cultivation of NK cells without genetic modifications. Many different protocols exist for *ex vivo* expansion of NK cells, all with different features and capacities. Here, we give a comprehensive overview of strategies to obtain appropriate amounts of functional NK cells. We will discuss starting material and culture systems as well as the use of cytokines, feeder cells, and other additives.

## Starting Material for NK Cell Expansion and Role of NK Cell Purity

Until recent, 92% of clinical studies used NK cells from peripheral blood, either donor (79% of recruiting trials) or patient derived (13% of recruiting trials) (36). Alternatives are the use of NK cell lines, or the differentiation of NK cells from umbilical cord blood or pluripotent stem cells (37–39). NK cell lines, such as NK-92, avoid the need for donor selection and enable the production of large cell doses to treat patients on a flexible schedule (40). Nevertheless, NK cell lines require growth inactivation mainly achieved by irradiation, possibly reducing their antitumor potential due to short *in vivo* persistence. Differentiation of NK cells from cord blood CD34^+^ cells is attractive because of the “off-the-shelf” availability from a cord blood bank. Similarly, NK cells from pluripotent stem cells are a promising concept for the future but still in early development (39, 41). In this overview, we focus on peripheral blood-derived NK cells, currently the main source for NK cells for clinical use.

The NK cell purity, meaning the frequency of NK cells among other cells, is an important factor for the intended therapeutic application. For *ex vivo* expansion, NK cells are often cultured within a mixture of cells, such as PBMC, thereby avoiding further purification. Whereas the cultivation of NK cells among other accessory cells is a practical strategy for autologous therapeutic settings, it may be critical for allogeneic applications, since non-NK cells may induce unwanted side effects. Alloreactive T cells are a major risk factor for the patient, as they mediate “graft-versus-host disease” (GvHD), a severe complication following allogeneic hematopoietic stem cell transplantation (HSCT) (42). Furthermore, donor-derived B cells can lead to B cell lymphoproliferative disorder after reactivation of an Epstein–Barr virus (EBV) infection (43, 44), and they can cause the passenger lymphocyte syndrome (45), both critical side effects for the patient. Therefore, purification of NK cells might be required and is realized so far in most clinical settings by magnetic cell separation, for instance, by depletion of CD3-expressing cells and subsequent enrichment for CD56-expressing cells (26, 46–49). In addition, a first proof of concept is shown for good manufacturing practice (GMP)-compliant fluorescence-activated cell sorting to purify for NK cell subsets, such as NK cells expressing a single KIR (50).

## Cytokine-Induced NK Cell Expansion

Aims of adoptive transfer of *ex vivo* expanded NK cells are the enhancement of natural cytotoxicity and homing to tumor sites under maintenance of “self” protection. Studies performed with cytokine-stimulated NK cells or PBMC have shown the safety of this approach and indicated some clinical responses upon adoptive NK cell transfer following HSCT. In the next paragraph, we summarize *ex vivo* NK cell expansion protocols starting with purified NK cells (Table 1) or PBMC (Table 2). Concepts administering cytokines in the presence of growth-inactivated feeder cells will be discussed in later sections of this article.

**Table 1:** ***Ex vivo* cultivation of pure natural killer (NK) cells with cytokines only**.

Protocol features	Starting material/culture system	NK cell expansion rate	NK cell purity	NK cell phenotype	NK cell function	Setting	Reference
IL-2 + IL-15	PBMC, CD3 depleted and CD56 enriched or PBMC, CD3/CD19 depleted in flasks	>1 (5 days)	75–100% NK ≤0.1% T cells	*Upregulated*: CD69, NKp30, and NKp44	Cytolysis of leukemia cell lines and primary acute leukemic blasts	*In vitro*	(51)
IL-2	PBMC, CD3 depleted, and CD56 enriched in bags and flasks	4–5 (12–14 days)	~92–95% NK <0.1% T cells	increased CD56^+^CD16^−^ frequency; increased p-STAT3 and p-AKT; increased lytic activity, upregulation of CD69, NKG2D, and natural cytotoxicity receptors (NCRs); increasing amount of NK cells without killer cell immunoglobulin-likereceptors	Improved cytotoxic activity against leukemia and tumors	Clinical	(26, 52, 53)
IL-15	Isolated CD3^−^CD56^+^ cells	N/A (2–5 weeks)	94–99% NK	Enhanced killing *via* NCRs, DNAM-1 and NKG2D	Enhanced cytolysis of lymphoma and rhabdomyosarcoma cell lines *via* NCRs	*In vitro*	(54)
IL-2 + IL-21	PBMC, sorted for CD3^−^CD56^+^	None with IL-21 only; strongly with IL-2 + IL-21	N/A	*Upregulated*: CD69, CD25; activation of STAT3	Enhanced cytotoxicity against K562	*In vitro*	(55)
IL-12 + IL-15 + IL-18	PBMC, sorted for CD3^−^CD56^+^ cells PBMC, CD3 depleted and CD56 enriched	N/A (12–16 h)	≥90% NK	*Upregulated*: CD94, NKG2A, NKp30, NKp44, NKG2D, NKp46, CD69, and CD25 *Downregulated*: NKp80	Memory: increased IFN-γ production upon stimulation that is preserved during cell division Responsive to picomolar concentrations of IL-2	*In vitro* Clinical	(56–59)

**Table 2:** ***Ex vivo* cultivation of natural killer (NK) cells with accessory cells**.

Protocol features	Starting material/culture system	NK cell expansion rate	NK cell purity	NK cell phenotype	NK cell function	Setting	Reference
IL-2	PBMC, CD3 depleted in bags and flasks	N/A (overnight)	33% NK 0.1% T cells	N/A	N/A	Clinical	(60)
		N/A (14–16 h)	26.7%		Enhanced cytotoxicity *In vitro*		(61, 62)
		N/A (overnight)	40% NK 0.9% T cells		Enhanced cytotoxicity *In vitro*		(43)
IL-15	PBMC, CD56 enriched	23 (20 days)	98% NK	Expression of NKp30, NKp44, NKp46, NKG2D, and 2B4	Cytotoxic *in vitro*	Clinical	(63)
IL-15 + IL-21	PBMC, CD3 depleted	3.7 CD56^+^/CD122^+^ (2–3 weeks)	>90% CD56^+^/CD122^+^ <0.3% CD3^+^/CD56^−^ <3% CD3^+^/CD56^+^	67% CD56^+^CD16^+^	Cytotoxic against K562 and patient bone marrow blasts	Clinical	(64)
OKT-3 + IL-2	PBMC in plates	193 (21 days)	~55% NK ~22% T cells	N/A	Substantial cytotoxicity against K562	*In vitro*	(65)
	PBMC in flasks	1,625 (20 days)	~65% NK ~22% T cells	*Upregulated*: 2B4, CD8, CD16, CD27, CD226, NKG2C, NKG2D, NKp30, NKp44, NKp46, LIR-1, KIR2DL3, and CXCR3 *Downregulated*: CCR7	Increased cytotoxicity against tumor cell lines and primary MM cells *In vitro*	*In vitro*	(25)
	PBMC	1,036 (total cells) (19 days)	~30% NK ~40% T cells	*Upregulated*: NKG2A, LILR-B1, NKG2D, NKp30, NKp44, and NKp46	*In vitro* cytotoxicity increases during culture	Clinical	(66)
	PBMC in a bioreactor, flasks, and plates	77—bioreactor 530—bags 770—flasks (20 days)	38%—bioreactor 31%—bags 44%—flasks	Bioreactor compared to flasks: higher expression of CD11b, NKG2D, and NKp44	Bioreactor compared to flasks: higher cytotoxicity	*In vitro*	(67)
OKT-3 + IL-2 + Alemtuzumab	PBMC in plates, flasks, and bags	646 (14 days) 1,537 (18 days)	60% NK 37% T cells <0.1% B cells	*Upregulated*: 2B4, NKG2D, NKp30, NKp44, KIR2DL1, LIR-1, and CD16 *Downregulated*: CCR7	Increased cytotoxicity *In vitro* and *in vivo*	Clinical	(68)
OKT-3 + IL-2 + IL-15	PBMC or CD56^+^ + CD56^−^ (1:1) in flasks and bioreactor (Cellbag)	PBMC: 112 1:1 Mix: 89 (21 days)	With PBMC: 34% With “1:1 Mix”: 92%	*Upregulated*: NKp30, NKp44, DNAM-1, NKG2D, and CD11a	Increased activity against neuroblastoma cell lines *in vitro* and *in vivo*	Preclinical model	(69)
aCD16 mAb + OK432 + IL-2	PBMC in flasks and bags	637–5,712 (day 21)	79% NK 8.4% T cells (day 21)	*Upregulated*: NKG2D, NKp44, and CD69 *Downregulated*: CD16 (transient)	Increased cytotoxicity against tumor cell lines and primary cancer cells *in vitro* ADCC activity	*In vitro*	(70)

## The Role of IL-2

Interleukin-2 plays an important role in activation of NK cells *via* binding to the IL-2 receptor (IL-2R), a heterotrimeric protein expressed on NK cells and other immune cells. This led to the interest in both (i) using IL-2 for stimulation of autologous NK cells in cancer patients and (ii) *ex vivo* activation and expansion of allogeneic donor NK cells for adaptive immunotherapy. At the beginning of the 1980s, researchers around Rosenberg and colleagues showed that IL-2 exposed lymphokine-activated killer (LAK) cells were able to attack autologous fresh tumor cells and that this effect could mainly be ascribed to NK cells (71, 72). Nevertheless, in first clinical trials using adoptive transfer of LAK cells and IL-2 therapy, the clinical response did not exceed the efficacy of IL-2 monotherapy (73).

Importantly, during the last 20 years, it has been elaborated that NK cells play a major role in the regulation of the balance between GvL and GvHD after allogeneic HSCT, especially haploidentical HSCT (33, 74–78), demonstrating improved anticancer activity while avoiding GvHD. In order to make haploidentical NK cells available for clinical use, large-scale GMP-conform manufacturing protocols were established. After starting with a leukapheresis product that was depleted for CD3^+^ cells and enriched for CD56^+^ cells, cultivation in medium containing IL-2 (1,000 U/mL) for up to 2 weeks yielded 0.1–3 × 10^9^ CD56^+^CD3^−^ NK cells (Table 1), sometimes sufficient for multiple infusions to patients with hematological malignancies (26). Median NK cell expansion was fivefold and median NK cell purity was >94 with <0.1% T cell contamination (26). *Ex vivo* stimulation with IL-2 induced elevated cytokine secretion by NK cells, enhanced intracellular STAT3/AKT signaling, and upregulation of various NCRs and NKG2D receptors (52). Depletion of CD3 cells from leukapheresis products without subsequent CD56 enrichment and short-term activation with IL-2 overnight led to a final product containing 40% NK cells (Table 2). In all cases, IL-2-activated NK cells demonstrated a much higher cytotoxic activity against K562 target cells compared to unstimulated NK cells (26, 43, 52, 53, 60). In addition, after cryopreservation and thawing, NK cells showed a moderate to high viability when activated with IL-2, whereas the viability of unstimulated NK cells was low (26).

Transfer into the clinic in 2004 and 2005 with first patient studies using those IL-2-activated donor NK cells were performed in parallel in Europe and the US, for both, haploidentical HSCT (53), and in the non-transplant setting (43). In the latter one, Miller and coworkers used IL-2 expanded haploidentical NK to treat 43 patients with advanced cancer (43), with 19 of them suffering from acute myeloid leukemia, followed by studies in patient with ovarian and breast cancer and B-cell non-Hodgkin lymphoma (60, 79). Importantly, the authors reported *in vivo* persistence and even expansion of the alloreactive donor NK cells in patients pretreated with high dose preparative regimen, consisting of 5 days of 60 mg/kg intravenous cyclophosphamide and 25 mg/m^2^ intravenous fludarabine (43). Of note, successful NK cell engraftment was dependent on the patients’ pretreatment regimen, which was also responsible for the patients’ elevated IL-15 plasma concentrations (43). In addition, it was demonstrated that *in vivo* persistence of donor NK cells at day 7 after infusion and successful *in vivo* expansion (more than 100 donor-derived NK cells per microliter of patient blood 14 days after transfer) correlated with leukemia clearance (60). Expansion of host regulatory T cells was associated with low numbers of NK cells (60). In parallel, Koehl et al. reported on three pediatric patients with multiply relapsed leukemia (still in blast persistence at HSCT) treated with repeated transfusions of IL-2-activated donor NK cells post-haploidentical HSCT (53), which led to complete remission remaining for several weeks up to some months. In the following clinical study, they also demonstrated a small clinical benefit in patients with various malignancies receiving IL-2-activated compared to patients receiving resting NK cells only (80). Interestingly, IL-2-stimulated NK cells but not unstimulated NK cells promoted NK cell trafficking and changes in the distribution of leukocyte subpopulations in the peripheral blood. In the meanwhile, safety and feasibility using IL-2-activated and -expanded NK cells for adaptive immunotherapy has been demonstrated in various clinical studies as summarized in a recent review by Koehl and others (33).

## Impact of IL-15 on NK Cell Expansion

Carson et al. postulated that NK cells might be dependent on other cytokines than IL-2 such as IL-15 (81, 82). The trimeric IL-15 receptor on NK cells shares two subunits with the IL-2R, but not CD25 forming the high affinity IL-2R. Therefore, they also share some functions, e.g., maintenance of NK cell survival (82). Similarities and differences between IL-2 and IL-15 effects on NK cells have been extensively reviewed elsewhere, and IL-15 might be the preferable cytokine for cancer therapy as it inhibits activation-induced cell death and it is considered safe (83–85). In addition, compared to IL-2, IL-15 leads to more sustained antitumor capacity of NK cells *via* signaling through mammalian target of rapamycin and stress-activated gene expression (86). However, recent data revealed that continuous IL-15 signaling causes functional exhaustion of NK cells by decreased fatty acid oxidation, resulting in lower cytotoxicity *in vitro* and decreased tumor control *in vivo* (87). Thus, optimal dosing and timing of IL-15 is critical for *ex vivo* NK cell activation. Purified NK cells expanded using IL-15 exhibit upregulation of NCRs and CD69 and cytolysis of leukemia and primary ALL blasts (51). Enhanced cytotoxicity of IL-15-stimulated NK cells against leukemia and rhabdomyosarcoma cell lines could be attributed to NCRs, DNAM-1 and NKG2D (54). Using IL-15 to expand NK cells from CD56-enriched PBMC for 20 days resulted in a 23-fold expansion of CD3^−^CD56^+^ NK cells with a final purity of about 98% (63). NK cells generated with the latter protocol were transferred to 15 non-small lung cancer patients in a phase I clinical trial in two to four doses of 0.2–29 × 10^6^ NK cells/kg, showing the safety of the approach (63).

## IL-21 Enhances NK Cell Effector Functions

The cytokine IL-21, in combination with IL-2 or IL-15, is utilized in some protocols for NK cell stimulation (55, 64). IL-21 belongs to the IL-2 family and signals through a heterodimer consisting of the common γ-chain and the IL-21 receptor α-chain. Activated CD4^+^ T cells are the main producers of IL-21 and IL-21 affects many different cell types expressing the IL-21 receptor, including NK cells (88). IL-21 plays a role in the development of NK cells from bone marrow progenitors (89), and, in mice, it dampens the expansion of NK cells but is required for functional NK cell maturation (90, 91). Recently, expansion of “memory-like” NK cells has been shown to be IL-21 dependent in the context of tuberculosis infection (92). Wendt et al. observed increased proliferation of CD56^bright^ human NK cells (55), but another group reported no effect of IL-21 on the proliferation of NK cells from healthy human donors and from HIV patients (93). Moreover, IL-21 is known to trigger apoptosis, resulting in a shorter lifespan of NK cells *in vitro* (90, 94). Thus, the time span NK cells are exposed to IL-21 appears critical (95, 96). Besides its effect on NK cell proliferation, IL-21 enhances the effector functions of NK cells, including secretory and cytotoxic functions as well as enhanced ADCC responses (93, 97, 98). Culturing CD3-depleted PBMC for 13–20 days with IL-21 and IL-15 without additional feeder cells yields activated NK cells with a purity of >90%, which were applied in a clinical trial with 41 leukemia patients receiving infusions of donor-derived NK cells 2–3 weeks after HSCT (64). Although the NK cells expanded weakly under this condition (3.7-fold), they possessed potent cytotoxic activity against primary bone marrow blasts prior to transplantation, and infusions with a median dose of 2 × 10^8^ NK cells/kg were well tolerated and correlated with a reduction in leukemia progression compared to historical controls (64).

## IL-12/15/18 Induced Memory NK Cells

Interleukin-12 was originally discovered as NK cell-stimulating factor, inducing proliferation, enhanced cytotoxicity, and production of IFN-γ by NK cells when added to PBMC (99, 100). IL-12 is produced by DCs, macrophages, and B cells, and its receptor consists of two subunits (α and β), which mediate signaling through members of the JAK–STAT family (101). IL-2 enhances the response of NK cells to IL-12 by increasing the expression of the IL-12 receptor and STAT4, a relevant factor for IL-12 signaling (102). Furthermore, it was revealed that IL-12-mediated IFN-γ production of NK cells requires priming with IL-18, a cytokine also known to enhance IL-15-induced NK cell proliferation (103, 104). Due to the synergistic effects, it seems reasonable to combine the different cytokines for *ex vivo* stimulation of NK cells. In this context, the combination of IL-12, IL-15, and IL-18 raised special interest, as it leads to the so-called “cytokine-induced memory-like” (CIML) NK cells in mice and humans, which exhibit an increased capacity to produce IFN-γ upon re-stimulation at later time points (56, 105). Importantly, this memory response is a cell intrinsic effect that is passed on to offspring cells and is maintained up to several months (56). In mice, the intrinsic ability for mediated IFN-γ production coincided with demethylation of the conserved non-coding sequence 1 in the IFN-γ locus (106). Furthermore, adoptive transfer of CIML NK cells had a clear antitumor activity against established melanoma or lymphoma *in vivo*, which required IL-2 from CD4^+^ T cells (57, 106). For both, murine and human NK cells, IL-12, IL-15, and IL-18 together induce an increased expression of CD25, making CIML NK cells responsive to low concentrations of IL-2 *in vitro* and *in vivo* (57, 58). Thus, there is a clear rationale to apply adoptive transfer of *ex vivo*-generated CIML NK cells together with IL-2 injections as a combination therapy. Recently, CIML NK cells together with low dose IL-2 therapy were evaluated in a first-in-human phase I clinical trial with promising results, as clinical response was observed in five of nine treated patients (107).

## Autologous Accessory Cells and Autologous Feeder Cells for NK Cell Expansion

Although cytokines efficiently activate NK cells and result in cell products with advanced effector functions, cytokines alone do not allow pronounced *ex vivo* expansion (Table 1). Consequently, in addition to the activation with cytokines, stimuli from autologous accessory cells can be used to further enhance the expansion of NK cells to overcome the hurdle of limited NK cell doses for adoptive NK cell therapy (Table 2). Outgrowth of NK cells from the whole PBMC fraction is more effective than cultivation of pure NK cells, because other cell types provide additional factors for NK cell proliferation. CD14^+^ cells, for instance, enhance the *ex vivo* NK cell proliferation *via* direct cell contact and soluble factors (108, 109). After activation, for instance by concanavalin A, T cells also trigger NK cell proliferation (110).

Stimulation of PBMC with IL-2 and the clinically approved anti-CD3 antibody OKT-3 leads to a profound outgrowth of NK cells (25, 65–67, 111), probably by activation of T cells and this is utilized by several clinical protocols for NK cell cultivation. Nevertheless, starting the culture from PBMC goes along with extensive coexpansion of unwanted CD3^+^/CD56^−^ T cells and CD3^+^/CD56^+^ NK-like T (NKT) cells, accounting for the majority of cells in the final cellular product. Surprisingly, infusion of this heterogeneous cell product without removal of potentially alloreactive T cells did not cause side effects, such as GvHD, in a safety trial with five cancer patients, evaluating the cultivated cellular product in an allogeneic setting (66). This can be explained by the fact that T cells may lose their alloreactivity during extended *ex vivo* expansion (112). Thus, low NK cell purities may be less critical for long-term cultivated cellular products compared to NK cells directly obtained from a donor, but more clinical data are required to prove this hypothesis. Of note, the approach also allows efficient expansion of functional patient-derived NK cells, as shown for B cell chronic lymphocytic leukemia and multiple myeloma patients, enabling therapy with autologous NK cells and further circumventing possible safety risks of therapy with donor-derived cells (25, 111).

Starting with PBMC enriched for CD56 cells together with the corresponding non-CD56 PBMC in a 1:1 mixture favors a 89-fold NK cell expansion with a final product consisting of 92% NK cells after 21 days (69). Alternatively, adding irradiated autologous PBMC to the culture is a strategy to benefit from these “feeder cells” for NK cell activation and expansion but to avoid their coexpansion (Table 3). Of note, to make a clear difference, we use the term “feeder cells” for all inactivated cells that are added to the culture, whereas cocultured non-NK cells that are not inactivated are defined as “accessory cells.” Besides its growth inactivating function, irradiation can induce upregulation of stress-regulated surface molecules on PBMC, such as ULBP1–3, that further trigger NK cell activation, e.g., through NKG2D (113). Still, irradiated autologous PBMC induce only weak NK cell proliferation without additional activation of the feeder cells (e.g., only 16-fold expansion within 2 weeks) (24). Whereas irradiated autologous PBMC previously activated with IL-2, OKT-3 and RetroNectin allow a median 4,720-fold NK cell expansion after 3 weeks with a NK cell purity of 91% starting from PBMC (114). To obtain a more pure final product with 98% NK cells, it is possible to start the culture with already CD3-depleted PBMC and add irradiated autologous PBMC as feeder cells together with IL-2 and OKT-3 (23). The highest purity can be achieved by cell sorting, representing also the method of choice to expand defined NK cell subpopulations. As demonstrated by Siegler et al., GMP-sorted and highly pure single KIR^+^ NK cells can be expanded 160- to 390-fold in 19 days with IL-2, IL-15, OKT-3, and irradiated autologous PBMC (50).

**Table 3:** ***Ex vivo* cultivation of natural killer (NK) cells with autologous feeder cells**.

Protocol features	Starting material/culture system	NK cell expansion rate	NK cell purity	NK cell phenotype	NK cell function	Setting	Reference
Irr. autologous PBMC (depleted for CD3^−^/CD56^+^ cells) + IL-2 + IL-15	PBMC, CD3 depleted, and CD56 enriched in flasks	16 (14 days)	97% NK 0.2% T cells	*Upregulated*: NKG2D, DNAM-1, NKp30, NKp44, CD158a, and CD158e	Efficient degranulation and lysis of K562 *In vitro*	*In vitro*	(24)
Irr. autologous PBMC activated with OK432, FN-CH296 and OKT-3 + IL-2	PBMC in flasks and bags	4,720 (21–22 days)	91% NK ~12% NK-like T and T	Strong expression of NKG2D and CD16	Elevated cytotoxicity that is maintained for up to 4 weeks after infusion to patients	Clinical	(114)
Irr. autologous PBMC + OKT-3 + IL-2	PBMC, CD3 depleted, and CD56 enriched in plates	169 (14 days)	84% NK	*Upregulated*: CD16, CD56, NKG2D, NKp30, and NKp44	Increased cytotoxicity against tumor cell lines *in vitro*	*In vitro*	(115)
	PBMC, CD3 depleted in flasks and bags	278–1,097 (21–26 days)	91–98% NK	Most cells express NKG2D, CD16, CD94, NKp46, KIR2DL1, KIR3DL1, and KIR2DL2/3	Efficient lysis of tumor cell lines *in vitro*; persistence in patients up to several months; cytotoxic potential is lost *in vivo*, while ability for ADCC is maintained	Clinical	(116)
	PBMC, CD3 depleted in bags	691 (14 days)	98% NK 0.06% T cells	*Upregulated*: NKG2C, NKp30, NK44, CXCR4, CD25, CD62L, and CD69	Increased cytotoxicity against tumor cell lines *in vitro*; antitumor effect and ADCC activity in a leukemia xenograft mouse model; up to 4 days persistence in patients	Preclinical model	(23)
		758 (14 days)	98% NK 0.4% T cells			Clinical	(117)
Irr. autologous PBMC (depleted for CD3^−^/CD56^+^ cells) + OKT-3 + IL-2	PBMC, CD3 depleted, and CD56 enriched in plates and flasks	546 (14 days)	94.9% NK 2.2% T cells	*Upregulated*: NKG2D, NKp30, NKp44, tumor necrosis factor-related apoptosis-inducing ligand, and DNAM-1 *Downregulated*: NKp80	Increased cytotoxicity against tumor cell lines *in vitro*	*In vitro*	(113)
Irr. autologous PBMC + OKT-3 + IL-2 ± IL-15	PBMC, CD3 depleted, and CD56 enriched in plates and bags	117/63 in bags (±IL-15) 993 in plates (19 days)	Bags: 45% NK 0.6% T cells	*Upregulated*: NKG2D, NKp44	High cytotoxicity against K562 and high productivity of IFN-γ	*In vitro*	(50)
	Good manufacturing practice killer cell immunoglobulin-like receptor (KIR) sorted NK cells in bags	160–390	~100% NK >0.01% T cells	Single KIR + NK cells	Anti-leukemic activity against primary acute myeloid leukemia cells *in vitro* and *in vivo*	Preclinical model	(50)

## NK Cell Expansion with Allogeneic Feeder Cells

Using irradiated allogeneic cells as feeder cells is another option to stimulate NK cell expansion *ex vivo* (118) (Table 4). Compared to autologous PBMC, allogeneic PBMC may be even more efficient as feeder cells for NK stimulation. Accordingly, in a study testing the expansion of NK cells from patients with advanced lymphomas or terminal solid tumors, 300-fold NK expansion was obtained with irradiated allogeneic PBMC feeder cells from healthy donors, whereas only 169-fold expansion was achieved with irradiated autologous PBMC feeder cells from the patients (115). Furthermore, whereas the availability of autologous feeder cells is limited, as they have to be obtained directly from the patient, for allogeneic feeder cells it is possible to utilize established cell lines. Cell lines can be grown easily to sufficient numbers and different cell lines in fact trigger NK cell proliferation, such as HFWT, K562, RPMI 1866, Daudi, KL-1, MM-170, and different EBV-transformed lymphoblastoid cell lines (EBV-LCL) (99, 119–122).

**Table 4:** ***Ex vivo* cultivation of natural killer (NK) cells with allogeneic feeder cells**.

Protocol features	Starting material/culture system	NK cell expansion rate	NK cell purity	NK cell phenotype	NK cell function	Setting	Reference
Irr. allogeneic PBMC activated with ConA + IL-2	*In vivo* IL-2 primed PBMC depleted for non-NK cells in flasks	1–148 (14 days)	64–98% NK	N/A	Cytotoxic activity against leukemic cell lines	Clinical	(123)
Irr. allogeneic PBMC activated with ConA, PHA and ionomycin + IL-2 + IL-15	PBMC, depleted for CD3, CD4, CD19, and CD33 in bags	80–200 (15 days)	91% CD56 0.3% CD3 (day 12)	*Upregulated*: CD16, CD25	Increased cytotoxicity against tumor cell lines *in vitro*; decreased frequency of INF-g producing cells	*In vitro*	(118)
Irr. allogeneic PBMC + OKT-3 + IL-2	PBMC, CD3 depleted, and CD56 enriched in plates	300 (14 days)	94% NK	*Upregulated*: CD16, CD56, NKG2D, NKp30, and NKp44	Increased cytotoxicity against tumor cell lines *in vitro*	*In vitro*	(115)
Irr. HFWT + IL-2	PBMC in flasks	113 (2 weeks)	86% CD56^+^/CD16^+^	N/A	Cytotoxic against tumor cell lines *in vitro*	Clinical	(124, 125)
Irr. Jurkat/KL-1 + IL-2	PBMC in flasks	~130 (2 weeks)	40–90% NK	*Upregulated*: CD54, CD11a, CD48, CD2, CD49d, CD58, NKp30, NKp44, 2B4, DNAM-1, NKG2D, CD25, and CD69 *Downregulated*: CD16	Increased cytotoxicity against tumor cell lines *in vitro* and antitumor activity *in vivo*	Preclinical model	(121)
Irr. K562 expressing membrane-bound IL-15 and 41BBL + IL-2	PBMC in plates	1,089 (3 weeks)	“Virtually pure”	N/A	N/A	*In vitro*	(126)
	PBMC in bags	23, 152, and 277 after 7, 14, and 21 days	96.8% NK 3.1% T cells (day 21)	Marked differences of gene expression profile compared to unstimulated or IL-2-stimulated NK cells	Increased cytotoxicity against tumor cell lines *in vitro* and antitumor activity *in vivo*	Preclinical model	(127)
	PBMC	447 (days 10–14)	88% NK 2.2% T cells (day 14)	Upregulated genes for cytolytic activity, cytokines, chemokines, activating receptors, adhesion molecules, cell cycle regulators, and multiple pathways	Increased cytotoxicity against primary MM cells *in vitro* and *in vivo*; high productivity of IFN-γ	Preclinical model	(128)
	PBMC in G-Rex, bags	442—G-Rex 227—bags (10 days)	70% NK 5–35% T cells	*Upregulated*: NKp30, NKp44, NKG2D, CD26, CD70, and CXCR3 *Downregulated*: CD16, CD62L	Increased cytotoxicity and ADCC against primary tumor cells *in vitro*; robust *in vivo* proliferation post-infusion	Clinical	(129, 130)
Irr. K562 expressing membrane-bound IL-15 and 41BBL + IL-15	PBMC, CD3 depleted, and CD56 enriched	1,000 (21 days)	N/A	*Upregulated*: CD56, NKG2D, tumor necrosis factor-related apoptosis-inducing ligand (TRAIL), CD158a, CD158b, and CD158e1	Increased cytotoxicity *in vitro* independent of killer cell immunoglobulin-like receptor mismatch; NK infusion contributed to acute graft-versus-host disease in first clinical trial	Clinical	(131, 132)
Plasma membrane particles of K562 expressing IL-15 and 41BBL + IL-2	PBMC in plates and flasks	1,265 (17 days)	86% NK cells 9% T cells 2% NK-like T	*Upregulated*: NKp30, NKp44, NKp46, NKG2D, 2B4, NKG2A, TRAIL, and Fas ligand (FasL) *Downregulated*: CD16	Increased cytotoxicity against leukemic cell lines and primary acute myeloid leukemia (AML) cells *in vitro*	*In vitro*	(133)
Irr. K562 expressing membrane-bound IL-21, 41BBL, CD64, CD86, and CD19 + IL-2	PBMC in flasks	4.8 × 10^4^ (21 days)	21.7% T cells	High expression of natural cytotoxicity receptors, CD16, and NKG2D	Cytotoxic against tumor cell lines *in vitro*; capable of ADCC; increased telomere length	*In vitro*	(134)
		2,363 (14 days)	83% NK 9.1% T cells	*Upregulated*: DNAM-1, NKG2D, CD16, and CD56	Cytotoxic and capable of ADCC against neuroblastoma cell lines *in vitro* and *in vivo*	Preclinical model	(135)
Plasma membrane particles of K562 expressing membrane-bound IL-21 and 41BBL + IL-2	PBMC	825 (14 days) >10^5^ (28 days)	>90% NK (day 14)	N/A	Increased cytotoxicity against leukemic cell lines and primary AML cells *in vitro*; enhanced proliferation *in vivo*	Preclinical model	(136)
Irr. allogeneic PBMC; irr. EBV transformed lymphoblastoid cell lines (EBV-LCL) (LAZ 388 cells) + PHA + IL-2	PBMC depleted for CD3 and monocytes in bags and plates	~43 (31–21 days)	90% NK <5% T cells	N/A	Increased cytotoxicity against tumor cell lines *in vitro*	Clinical	(137, 138)
Irr. EBV-LCL (TM-LCL) + IL-2	PBMC, CD3 depleted, and CD56 enriched in bags	800–1,000 (2 weeks)	98% NK	*Upregulated*: TRAIL, FasL, NKG2D, NKp30, NKp44, NKp46, CD48, CD25, LTB, MX1, and BAX	Increased cytotoxicity against tumor cell lines *in vitro*	*In vitro*	(139, 140)
Irr. EBV-LCL (SMI-LCL) + IL-2	PBMC, CD3 depleted, and CD56 enriched in bags	3,637 (24–27 days)	99.7% NK			Clinical	(141)
	PBMC, CD3 depleted, and CD56 enriched in CliniMACS Prodigy	850 (14 days)	>99% NK	*Upregulated*: TRAIL, FasL, NKG2D, NKp30, NKp44, and DNAM-1	Increased cytotoxicity and ADCC against tumor cell lines *in vitro*	*In vitro*	(142)
Irr. EBV-LCL (SMI-LCL) + IL-2 + IL-21	PBMC depleted for non-NK cells (research kit) in plates and flasks	2,900 (14 days) 2.7 × 10^11^ (46 days)	>99% NK	*Upregulated*: TRAIL, NKG2D, and DNAM-1	Cytotoxic against tumor cell lines *in vitro* and *in vivo*; enhanced and sustained production of IFN-γ and TNF-α	Preclinical model	(96)
Lysate of CTV-1	PBMC, CD3 depleted, and CD56 enriched	N/A (overnight)	97–98% NK	*Upregulated*: CD69 *Downregulated*: CD16	Cytotoxic against NK-resistant leukemia cell lines and primary tumors *in vitro*	Clinical	(143, 144)

Culturing PBMC together with the Wilms tumor cell line HFWT and IL-2 leads to significant NK cell expansion (124, 145), and interestingly under this condition NK cells not only arise from mature CD3^−^CD56^+^ NK cells but also from CD3^−^CD14^−^CD19^−^CD56^−^ NK cell precursors expressing CD122 (146). In 2004, early clinical data showed that adoptive transfer of autologous NK cells generated by coculture with irradiated HFWT is safe and patients with recurrent malignant glioma partially responded to the treatment (125).

Another advantage of cell lines is that it is relatively easy to genetically modify them and to integrate additional factors for NK cell stimulation. In recent years, modified K562 cells have been utilized, such as K562 expressing membrane-bound IL-15 and 41BBL (K562-mb15-41BBL) (126). While unmodified K562 only induce a weak NK cell proliferation (2.5-fold NK cell expansion in 1 week), with K562-mb15-41BBL the NK cell number can be significantly increased by 20- or 1,000-fold in 1 or 3 weeks (126). In addition, stimulation of NK cells with K562-mb15-41BBL demonstrated that NK cells actually have a substantial proliferative potential *ex vivo*, with up to 30 population doublings and 5.9 × 10^4^-fold NK cell expansion (147). NK cells expanded with K562-mb15-41BBL exhibit enhanced natural cytotoxicity against several allogeneic and autologous tumors *in vitro*, efficiently mediate ADCC and showed antitumor efficacy in mouse xenograft models for the treatment of sarcoma and myeloma (128, 148, 149). Of note, in a clinical trial assessing adoptive transfer of K562-mb15-41BBL following HSCT, acute GvHD occurred in five of nine patients, although the donors were completely HLA matched and the doses of injected NK cells and cotransferred T cells were low (1–10 × 10^5^ and ≤2 × 10^4^/kg) (131). These observations suggested that the acute GvHD was T cell mediated, but NK cells apparently may promote this severe side effect indirectly (150). Importantly, another group utilized NK cells expanded with a similar K562 variant expressing 41BBL and IL-15 in another treatment setting and did not observe GvHD, although up to 1 × 10^8^ NK cells/kg were administered (129).

Furthermore, Denman and colleagues revealed that K562 expressing 41BBL and membrane-bound IL-21 instead of IL-15 are even more effective for *ex vivo* expansion of NK cells, and weekly restimulation with this cell line supports a sustained NK cell proliferation over several weeks (134). In coculture with K562 expressing membrane-bound IL-21 and 41BBL, NK cells show an increased telomere length and enhanced activation of the STAT-3 signaling pathway, explaining the positive effect for sustained expansion of NK cells over long time (134, 151). Adoptive transfer of NK cells expanded with K562 expressing membrane-bound IL-21 and 41BBL into tumor-bearing mice improved the survival of the animals, indicating a therapeutic effect of these NK cells (135).

The stimulatory effect of EBV-LCL on NK cell proliferation was discovered more than 30 years ago (152). In 1994, an early clinical trial already evaluated the adoptive transfer of autologous NK cells expanded with the LAZ 388 cell line to treat 10 patients with metastatic renal cell adenocarcinoma (137). More recently, the cell lines TM-LCL and SMI-LCL were reported for NK cell expansion, allowing around 800-fold expansion of highly pure NK cells within 2 weeks (139–142). NK cells generated with these EBV-LCL feeder cells are currently applied in a study testing them for adoptive transfer in an autologous setting with intended doses up to 1 × 10^9^ NK cells/kg (141). Recently, it was reported that repeated stimulation with SMI-LCL in IL-2-containing medium and adding IL-21 only at start of cultivation enables 10^11^-fold NK cell expansion after 6 weeks, to our knowledge representing the most efficient protocol to expand NK cells at the moment (96). NK cells generated with the latter method are highly cytotoxic *in vitro*, show a sustained high productivity of IFN-γ and TNF-α, similar to CIML NK cells, and they efficiently controlled melanoma in a xenograft mouse model (96).

Although feeder cells, and allogeneic feeder cell lines in particular, make it possible to generate substantial numbers of NK cells for adoptive therapy, from a regulatory point of view this strategy has drawbacks as feeder cell lines must be qualified as safe for human use. The cell line qualification of modified K562 cells, for instance, includes costly viral testing and assays to prove absence of bacterial and *Mycoplasma* contamination (153). In this context, lysates from cell lines containing the NK cell-stimulating factors could be an alternative to the intact feeder cells to minimize regulatory concerns. It was demonstrated that short cultivation of NK cells with lysate of the leukemia cell line CTV-1 primes NK cells to specifically lyse cell lines that are resistant to resting NK cells (143). Interestingly, the priming effect of CTV-1 on NK cells is KIR independent and does not require supplementation of cytokines, such as IL-2 or IL-15, making this an unique approach for NK cell activation (154). NK cells primed with CTV-1 were evaluated in the first UK clinical trial of a cell therapy regulated as a medicine, with an anti-leukemia effect in four of seven treated patients and no evidence of NK cell infusion-related toxicities (144). Another step forward from a regulatory standpoint could be to add only specific fragments of feeder cells to the culture that are responsible for the desired NK cell activation, instead of using intact feeder cells or their lysates. Of note, NK cells can be expanded *ex vivo* with IL-2 and plasma membrane particles prepared from K562-expressing membrane-bound IL-15 and 41BBL with a rate of expansion that is comparable to stimulation with intact feeder cells and far better than stimulation with soluble IL-15, 41BBL, and IL-2 (133). Plasma membrane particles from K562 expressing membrane-bound IL-21 and 41BBL work for *ex vivo* NK cell expansion as well and may be an option for *in vivo* NK cell expansion, as demonstrated in a first proof of concept using a mouse model (136).

## Technical Aspects of NK Cell Expansion

In general, one encounters technical challenges and opportunities when manufacturing NK cells as medicinal products, as reviewed recently (155). In this section, we focus on technical options for NK cell culture, ranging from simple cell culture plates for small scale experiments to highly standardized and automated systems for clinical scale. The selection of the adequate culture system is based on the intended application of the cells. Most preclinical experimental studies grow NK cells in cell culture plates or tissue culture (T) flasks. These are commonly used and very convenient to test and compare different culture additives in parallel, e.g., different cytokine concentrations. However, for clinical applications in large scale, cultivation in plates and flasks is rather inappropriate for different reasons. First, due to the small volume of T flasks, numerous T flasks have to be handled at the same time, with for instance 51 T flasks for the treatment of a single patient (116). In addition, T flasks have to be opened from time to time for medium exchange or harvesting of cells, bearing the risk of contaminating the cellular product. Although the likelihood of contamination for each T flask is reduced to a minimum by sterile workflows in safety cabinets, the remaining risk potentates by the number of flasks.

To overcome the drawbacks of small cell culture vessels, clinical NK cell cultivation is often done in cell culture bags, which make it possible to culture high volumes in a closed system, as all required steps can be done by sterile welding of tubing connections for the transfer of media, harvesting of cells, etc. Unfortunately, different reports describe that the NK cell expansion performance is reduced after transition of a protocol from T flasks to larger scale in cell culture bags (50, 67). In addition, bag systems still require several labor-intensive interventions during the culture, especially when different cultures are set up in parallel.

The G-Rex vessel is another system avoiding frequent processing steps for exchange of medium during the culture. In contrast to normal cell culture flasks, the bottom of the G-Rex is highly gas permeable, ensuring optimal CO_2_ exchange and O_2_ supply for the cells. Thus, by its design, G-Rex flasks can be filled directly with a high level of cell culture medium and exchange of medium is not necessary for long time. For NK cell culture, G-Rex were used for example for 10 days of culture without any cell manipulation or feeding, and resulted in higher fold expansion of NK cells compared to cell culture bags (130). Unfortunately, although G-Rex are scalable in general, multiple G-Rex flasks are still required to achieve high cell numbers for clinical trials, which can be cumbersome and costly, and G-Rex flasks are still an open system and may require adaption to a closed system (156).

Automated systems combine the need for reduced interventions during the culture with a closed system. Automation of the cell manufacturing ensures constant product quality without the need for highly skilled experts, is finally cost saving, and may be required for cellular therapy to become available beyond specialized academic centers (157). Although early integration of automation is associated with higher capital costs in the development phase, it allows a smooth transition at later stages of clinical development (158). A first feasibility study of automated NK cell cultivation with a stirred bioreactor was already published in 1996, showing advantages of the bioreactor culture over manually handled controls (159). More recently, different investigators report automated NK cell expansion procedures with a rocking motion bioreactor (67, 69, 156, 160), yielding 2–10 × 10^9^ NK cells under GMP-compliant conditions. However, the latter system still needs preceding manual cultivation, because relatively high cell numbers are required as inoculum for the automated culture (67, 69, 156, 160). Alternatively, fully automated NK cell expansion with an automated cell processing device can be performed for clinical use, with as little as 10^6^ NK cells being sufficient to initiate the automated culture that can yield up to 2.7 × 10^9^ NK cells after stimulation with clinical grade feeder cells (142). Of note, in addition to the culture process, the cell processing device is designed for GMP-compliant cell separation, concentration, and washing applications, so that combined NK cell purification, cultivation, and final formulation of the cellular product is possible fully automated (161). Thus, the whole processing, from the starting material, such as a leukapheresis product, to the finally expanded NK cells, readily prepared for infusion, can be covered by a single instrument.

Centralized processing of NK cell products probably will be carried out mainly in specialized centers for manufacturing of cellular products. Consequently, after *ex vivo* cultivation, storage of the NK cell product and shipment to the location of use will be needed. Compared to naive NK cells, IL-2-activated NK cells are less sensitive to freezing, as they show higher recovery and viability after thawing (26). Still, different groups state that cryopreservation of cultivated NK cells goes along with a drop in cell viability and cytotoxicity, whereas the latter can be restored by a short re-stimulation, e.g., by a short resting in IL-2-containing medium (139, 156). Poor survival of the NK cells can be an issue during further *in vitro* culture post thawing, so that shipping of freshly formulated cells for direct infusion may be advantageous (129). Interestingly, some groups recently claim that freezing and thawing does not influence the cytotoxicity or the proliferative ability of cultivated NK cells in their hands (24, 68). These divergent observations possibly result from different cultivation methods and different protocols for freezing and thawing, which should be investigated further. Without freezing, transport of the readily prepared cells in an appropriate time frame is challenging, and any delay during the shipment affects the quality of the cellular product with critical consequences for the patient. Alternatively, automated and closed systems for cell processing open the way for scale out strategies and de-centralized NK cell manufacturing directly at the location of intended use, avoiding the freezing and shipment process (142). But, although de-centralized manufacturing in the clinics seems promising, cellular therapeutics are very complex and still in early development, so that manufacturing by well-trained specialists in specific facilities is reasonable at that state.

## Regulatory Aspects of NK Cell Cultivation for Clinical Use

Apart from technical difficulties, one has to consider regulatory aspects for the use of *ex vivo*-generated NK cells with regulations varying in time and geographical policies (153). In Europe, for instance, cytokine-activated and -expanded NK cells are currently classified as advanced therapy medicinal products and will be regulated accordingly either centralized or under the hospital exemption by the member states [Regulation (EC) No 1394/2007; Directive 2001/83/EC and Regulation (EC) No 726/2004]. Quality aspects related to somatic cell therapy medicinal product as defined in guidelines (CPMP/BWP/3088/99; EMEA/CHMP/410869/2006; Ph. Eur. 0784: Ph. Eur. 5.14) will apply to the identity, potency, and activity. The establishment of correspondingly adequate in process and quality controls as well as of process target values and product specifications will have to take into account the variability of the primary effector cell as the starting material (162).

## Conclusion and Outlook

Comparing different protocols for NK cell cultivation in detail is challenging as these are extremely heterogeneous. The duration of *ex vivo* NK cell cultivation ranges from a few hours for short NK cell activation up to several weeks for long-term expansion, different starting materials are in use with varying NK cell purities, different cytokines are combined at different doses, and NK cells often are cocultured with different feeder cells at different NK-to-feeder ratios. Nevertheless, overall differently *ex vivo* expanded NK cells exhibit some common characteristics.

In general, *ex vivo* cultivated NK cells show an increased cytotoxicity and may become even responsive against tumor targets previously appearing resistant to NK cell lysis. This explains the use of IL-2 or IL-15 in virtually every protocol, as it is known since a long time that both cytokines amplify NK cell activity (81, 163). However, upon NK cell activation with different stimuli, including IL-2 and IL-15, downregulation of CD16 surface levels occurs by metalloproteases-mediated shedding of CD16 (164–166). The Fc receptor CD16 is crucial for NK cells to perform ADCC and would be of particular importance for potential combination therapies using NK cells together with therapeutic antibodies. Of note, although reduced levels of CD16 on NK cells are observed for several NK cell cultivation protocols the NK cells still mediate ADCC (70, 129, 142). Nevertheless, inhibition of the relevant metalloproteases to maintain CD16 on NK cells could be an option to further increase the ADCC function of *ex vivo* activated NK cells (164, 167).

Another clinically highly relevant aspect is the tumor-induced immunosuppression as important challenge for all cell therapeutic strategies. Remarkably, it ruled out from most preclinical and clinical NK cell studies that NK cells may gain the capability to overcome tumor immunosuppression. Different research groups have reported signs of NK cell suppression in cancer patients such as a lower expression of NK cell receptors, e.g., NCRs, NKG2D, DNAM-1, and 2B4 (22, 25, 168–170), the shedding of tumor cell ligands, such as NKp30 and NKG2D (171–174), or the release of blocking NKG2D ligands, such as MICA and ULBP3, *via* tumor-derived exosomes (175, 176). Notably, *ex vivo* cultivation of patient-derived NK cells is often possible with same efficacy as for donor-derived NK cells (25, 111) and can normalize the NK cell phenotype and activation (25). Additionally, elevated levels of NKG2D on *ex vivo*-activated NK cells can scavenge shed NKG2D ligands and counter their inhibitory effect (177). Furthermore, the high cytotoxicity of *ex vivo* expanded NK cells has been shown to be independent of KIR inhibition for some protocols (107, 132).

In comparison to other cell therapeutic approaches using, e.g., T cells, donor-derived allogeneic NK cells mediate GVL without an elevated risk for GVHD or even with a GVHD-reducing effect, as reported in mice and men (74, 75, 77, 78). However, contradictory results regarding GVHD induction have been reported in clinical trials assessing adoptive transfer of NK cells expanded with K562 feeder cell variants expressing 41BBL and IL-15 (129, 131). These reports show that there are still open questions that have to be unraveled to better understand the complex role of NK cells and their specific subsets in the bidirectional regulation of GVL and GVHD.

In conclusion, many different protocols are in use to expand NK cells *in vitro*, each with its specific advantages and disadvantages in regard of cell numbers, function, and handling efforts. The data summarized in this review underline the complexity related to the design of an optimal NK cell therapeutic protocol that should be not only reliable and safe in use but also highly efficient in targeting different forms of malignancies. With this in mind, additional studies need to be envisioned that not only further address *ex vivo* NK cell purification, expansion, and activation strategies but also the final clinical setting including pre-conditioning, dosing, and timing of the NK cell application. Efforts for harmonization of protocols at the European and worldwide level should be undertaken to ensure highest quality and efficacy of the NK cell product for clinical application. Finally, with regard to the possible tumor-mediated immunosuppression, therapeutic concepts have to be developed that either directly strengthen NK cells to deal with the hostile tumor environment and/or specifically counteract tumor-induced immunosuppressive mechanisms.

## Author Contributions

MG, JW, and EU extensively reviewed the current literature on *ex vivo* NK cell cultivation and expansion and prepared a comprehensive overview that is listed in the tables. MG, JW, UK, AC, VH, and EU wrote and critically reviewed the manuscript.

## Disclaimer

MG and VH were employed by company Miltenyi Biotec.

## Conflict of Interest Statement

All authors, including MG and VH, declare that they have no commercial, proprietary, or financial conflict of interest.
